# PARP inhibition prevents escape from a telomere-driven crisis and inhibits cell immortalisation

**DOI:** 10.18632/oncotarget.26499

**Published:** 2018-12-25

**Authors:** Greg Ngo, Sam Hyatt, Julia Grimstead, Rhiannon Jones, Eric Hendrickson, Chris Pepper, Duncan Baird

**Affiliations:** ^1^ Division of Cancer and Genetics, School of Medicine, Cardiff University, Heath Park, Cardiff, UK; ^2^ Department of Biochemistry, Molecular Biology, and Biophysics, University of Minnesota, Minneapolis, MN, USA; ^3^ University of Sussex, Brighton and Sussex Medical School, Brighton, UK

**Keywords:** telomere, genome instability, PARP1, crisis, cancer

## Abstract

Telomeric crisis is the final replicative barrier to cell immortalisation; it is characterised by genome instability and cell death and is triggered when telomeres become critically short and are subjected to fusion. Pre-cancerous lesions, or early stage cancers, often show signs of a telomere crisis, suggesting that escape from telomere crisis is a prerequisite for disease progression. Telomeric crisis therefore represents an attractive, and as yet unexplored, opportunity for therapeutic intervention. Here, we show that two clinically approved PARP inhibitors, selectively eliminate human cells undergoing a telomere-driven crisis. Clonal populations of a colorectal cancer cell line (HCT116), or the plasma cell leukaemia cell line (JJN-3), expressing a dominant-negative telomerase, entered a telomere-driven crisis at defined population doubling points and telomere lengths. The addition of the PARP inhibitors, olaparib or rucaparib prevented these cells from escaping crisis. PARP inhibition did not alter cellular proliferation prior to crisis, rates of telomere erosion or the telomere length at which crisis was initiated, but affected repair of eroded telomeres, resulting in an increased in intra-chromosomal telomere fusion. This was accompanied by enhanced DNA damage checkpoint activation and elevated levels of apoptosis. We propose that PARP inhibitors impair the repair of dysfunctional telomeres and/or induce replicative stress at telomeres to inhibit escape from a telomere crisis. This is the first demonstration that a drug can selectively kill cells experiencing telomeric crisis. We propose that this type of drug, which we term ‘crisolytic’, has the potential to eliminate pre-cancerous lesions and tumours exhibiting short dysfunctional telomeres.

## INTRODUCTION

Telomeres are nucleoprotein structures that protect the ends of linear eukaryotic chromosomes. In humans, telomeres are composed of single and double-stranded TTAGGG repeats that are bound by the telomere capping complex, Shelterin [1]. The primary function of telomeres is to prevent the activation of the DNA damage response by shielding the end of chromosomes from being recognised as DNA strand breaks [2]. Due to the end replication problem, telomeres in most somatic cells in humans become progressively shortened with each cell division. The process of telomere erosion eventually leads to sequential activation of two cellular states called senescence and crisis, which limit cell proliferation [3]. Senescence is a cell cycle arrest state, which is triggered when short telomeres partially lose their end-protective function and activate the DNA damage checkpoint machinery [4]. This proliferative block can be bypassed by mutations in the TP53 pathway, which permits further cell division and telomere erosion [5]. This, in turn, leads to the onset of crisis, which is characterised by genome instability and cell death and is triggered when telomeres become critically short and are subjected to DNA repair activity resulting in telomere fusion [6].

Genome instability induced during telomere crisis is considered to drive clonal evolution and cancer progression [1]. Pioneering studies using telomerase null mice showed that telomere crisis induces non-reciprocal translocations that promote cancer progression in these mice [7, 8]. Telomere erosion and crisis are associated with the initiation of various malignancies in humans [9–13]. Furthermore, the telomere length of tumour cells can accurately predict the progression of both solid and haematological cancers [14–16]. The clear requirement for cells to escape from telomere crisis and establish replicative immortality during tumour progression is supported by the finding that all cancer cells activate telomerase, or the alternative lengthening of telomeres (ALT) pathway, to facilitate ongoing proliferation [3, 17]. These findings indicate that interventions that could modulate the ability of cells to escape a telomeric crisis, or specifically sensitize cells with short telomeres to therapy, could represent a promising alternative treatment strategy in the earliest stages of tumour progression.

HCT116 cells (a human colorectal cancer cell line) undergoing a telomeric crisis require the activity of DNA ligase III (LIG3), but not DNA ligase IV (LIG4) to escape a telomere crisis [18]. The reason for this requirement is not completely understood, but LIG3 is believed to be needed for alternative non-homologous end-joining (A-NHEJ), whereas LIG4 promotes classical non-homologous end-joining (C-NHEJ) [19]. PARP1, a DNA repair protein that catalyses the process of poly-ADP-ribosylation (PARylation), promotes A-NHEJ with LIG3 [20, 21]. PARP1 is recruited to various types of DNA damage and acts by cleaving NAD+ to nicotinamide and ADP-ribosyl moieties, which are successively used to synthesise covalently linked ADP-ribose chains on target proteins [22].

PARPi (PARP inhibitors) are a class of small molecule inhibitors developed to inhibit the process of poly-ADP-ribosylation by PARP1. Most PARPi are nicotinamide mimics that bind into the NAD+ pocket of PARP1 thereby inhibiting its PARylation activity [23]. Importantly, PARPi potentiate the cytotoxicity of various chemotherapeutic agents suggesting that their mechanism of action is distinct and complementary to existing treatments [23]. In 2005, two landmark studies showed that PARPi selectively killed breast cancer allele 1 or breast cancer allele 2 (BRCA1/BRCA2)-deficient cancer cells by inducing the collapse of replication forks [24, 25]. This finding paved the way for PARPi to be used as a monotherapy agent to kill BRCA1/BRCA2-deficient cancer cells and other cancerous cells that share molecular features with BRCA1/BRCA2-deficient tumours [26]. Following some positive clinical trials, two PARPi, rucaparib and olaparib, have been clinically approved for the treatment of ovarian cancer [23].

In this study, we examined whether PARPi affect the ability of human cells to escape a telomere crisis. We found that two clinically approved PARPi, olaparib and rucaparib, prevent human colorectal cancer cells and cancerous plasma cells from escaping telomere crisis and establishing replicative immortality. Our data indicate that PARPi selectively kills cells undergoing a telomere crisis by affecting the repair of eroded telomeres and thus increasing DNA damage and apoptosis.

## RESULTS

### Olaparib and rucaparib inhibit HCT116 dominant negative human telomerase (DN-hTERT)-expressing cells from escaping a telomere crisis

To study the effect of PARP inhibition during a telomere-driven crisis, we used a TP53-positive HCT116 colorectal cancer cell line expressing a DN-hTERT construct. Telomerase activity is abrogated in these cells and they show progressive telomere erosion as a function of cell division. After 55 population doublings (PDs) from the point of single-cell cloning, these cells entered a telomere erosion-induced crisis-like state, characterised by slowed cell growth, increased apoptosis and the induction of telomere fusion events [18]. After approximately 30 days in crisis, the HCT116 DN-hTERT clones reproducibly escaped crisis and become (re)-immortalised, following the re-establishment of telomerase activity, the lengthening of telomeres and the cessation of telomere fusions [18].

**Figure 1:**
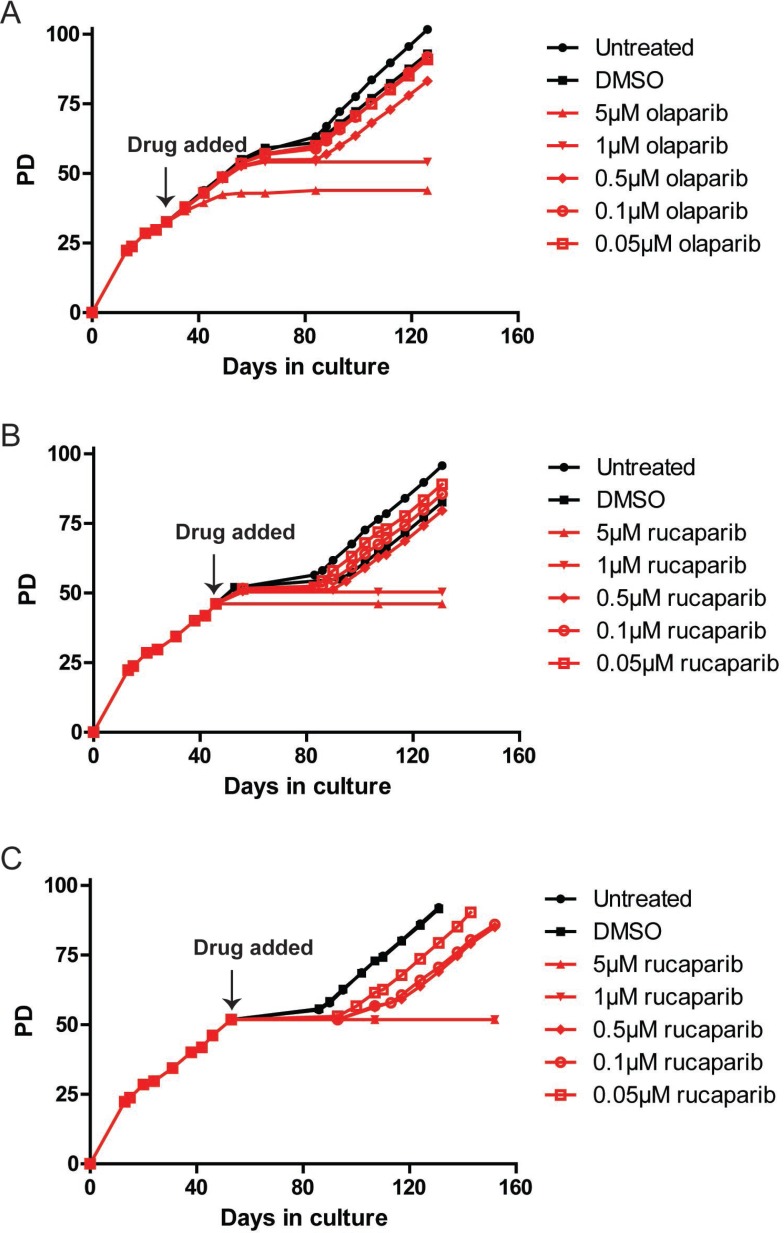
PARPi prevent HCT116 cells from escaping telomere crisis (**A**–**C**) Growth curve of HCT116 DN-hTERT cells treated with the indicated concentrations of olaparib and rucaparib (PD = population doubling). Frozen HCT116 DN-hTERT cells at a required PD were thawed and propagated before each PARPi was added at the time indicated. Media/drug were replaced at least once every three days until the termination of the experiments.

To understand whether PARPi could affect the ability of an HCT116 DN-hTERT clone to escape a telomere crisis, we cultured these cells in a range of concentrations of olaparib (0.05 µM, 0.1 µM, 0.5 µM, 1 µM and 5 µM) starting at PD33 — 22 PDs before these cells were expected to enter crisis. We chose this concentration range because at least 1 µM of olaparib is required to fully inhibit PARP1 activity in HCT116 cells [27]. Another previous study showed that 0.4 µM of rucaparib effectively inhibited PARP activity in SW620 colorectal cancer cells for up to 3 days [28]. Thus, to ensure that PARP1 remained inhibited, new drug was added to the cells every 3 days. As controls, we also examined untreated cells and those treated with dimethyl sulfoxide (DMSO, the vehicle). Consistent with our previous findings [18], untreated HCT116 DN-hTERT cells (PD33) underwent an additional 22 PDs before entering crisis, characterised by slow cell growth and the appearance of large vacuolated cells (Figure 1A, data not shown). After approximately 30 days in crisis, some cells escaped crisis and continued to divide at the same rate as that observed prior to crisis, continuing to over 100 PD when the experiment was intentionally terminated.

Cells treated with DMSO or low concentrations of olaparib (0.05 µM, 0.1 µM and 0.5 µM) behaved similarly to the untreated cells, as these cells entered crisis at about the same time (PD55, day 56) and all managed to escape with the 0.5 µM-treated cells lagging slightly behind the lower concentrations and controls (Figure 1A). Cells treated with 1 µM of olaparib initially behaved similarly to the control cells, dividing at the same rate and entering crisis at the same PD point. In striking contrast however, despite keeping these cultures for up to 126 days, all of the cells eventually died and (per force) no cells managed to escape crisis (Figure 1A). Moreover, cells treated with 5 µM olaparib only managed to divide up to PD44 but then stopped dividing and died.

To confirm the effect of PARPi on the escape from a telomere-driven crisis, we tested an additional PARPi, rucaparib. We examined whether a shorter exposure to PARPi could prevent escape from crisis by adding rucaparib to HCT116 DN-hTERT cells at a time point closer to the onset of crisis. To do this, we added the same range of concentrations of rucaparib to HCT116 DN-hTERT cells at PD46 and PD52 (9 and 3 PDs prior to crisis, respectively; Figure 1B, 1C). It was clear from these data that even short-term exposure of HCT116 DN-hTERT cells to high concentrations of rucaparib (1 µM and 5 µM) was also sufficient to prevent these cells from escaping a telomere-driven crisis (Figure 1B, 1C). We concluded that treatment of HCT116 DN-hTERT cells with PARPi prior to the point at which the cells enter crisis was sufficient to prevent these cells from escaping crisis.

We next considered whether the effect of olaparib and rucaparib on the ability of HCT116 DN-hTERT cells to escape crisis was specifically a consequence of telomere erosion, or whether this effect might have arisen because of impaired cell growth kinetics in the long-term presence of PARPi. To assess this, we tested how both olaparib and rucaparib affected the growth rates of HCT116 cells that did not express DN-hTERT (Supplementary Figure 1). We found that only cells treated with 5 µM of olaparib or rucaparib exhibited slightly slower growth (0.74 PD/day and 0.80 PD/day, respectively) than all other treatment conditions and controls (untreated controls = 0.96 PD/day), but even these cells managed to propagate for over 52 PD at which point all the cultures were intentionally terminated (Supplementary Figure 1). We therefore concluded that high dose and short-term exposure of PARPi can selectively eliminate HCT116 DN-hTERT cells undergoing a telomere-driven crisis and prevent these cells from becoming immortalised and that this effect is not related to a general inhibition of proliferation.

### Rucaparib and olaparib inhibit JJN3 DN-hTERT cells from escaping a telomere crisis

**Figure 2:**
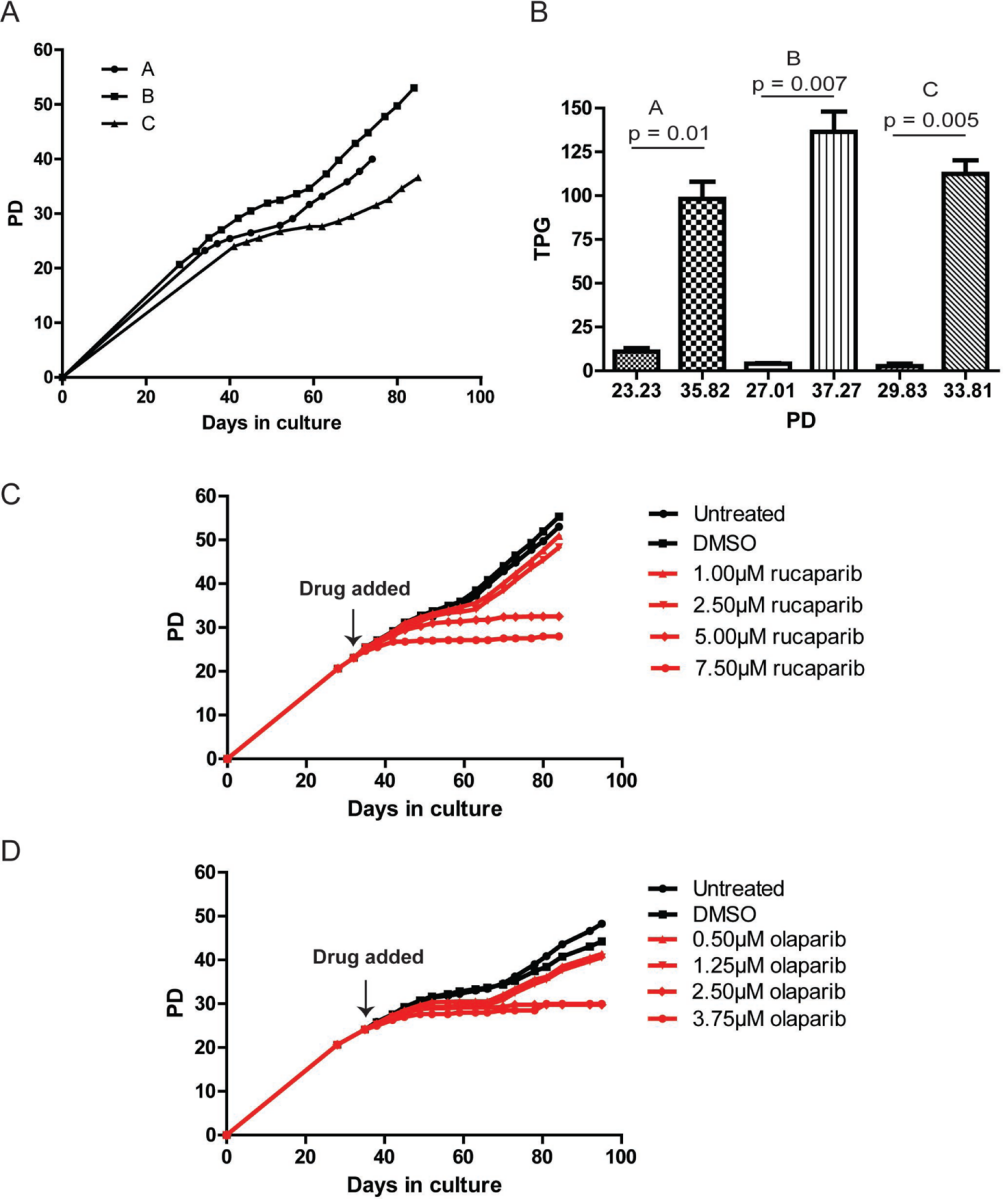
PARPi prevent JJN-3 cells from escaping telomere crisis (**A**) Growth curve of three clonal populations of JJN-3 cells expressing DN-hTERT (PD = population doubling). (**B**) Telomerase activity of each JJN-3 clonal population was monitored before and after crisis and plotted as total product generated (TPG). Significance was determined using a paired *t*-test. (**C**, **D**) Growth curve of JJN-3 DN-hTERT cells treated with the indicated concentrations of olaparib and rucaparib. Each PARPi was added at the time indicated, and media/drug replaced at least once every three days until the termination of the experiments.

To further test the effect of PARPi on telomere crisis, we examined the p53-null multiple myeloma cell line, JJN-3 [29]. To induce a telomere crisis, we transfected JJN-3 cells with a DN-hTERT construct and analysed how this affected the proliferation of three individual JJN-3 clones (Figure 2A). After 25 to 30 PDs, each clone experienced a period of stalled growth, before eventually recovering and growing steadily until the experiments were terminated (Figure 2A). We next examined telomerase activity in these clones and found that while telomerase activities were low before the slow growth period, all three clones had significantly higher activity of telomerase after they had recovered, indicating that these cells had been re-immortalised (Figure 2B). Examination of telomere length distributions using STELA and telomere fusion using single-molecule PCR [30] confirmed that JJN-3 DN-hTERT cells had short telomeres and increased telomere fusions during the slow growth period, but that their telomeres were elongated and stabilized (as shown by a reduction of telomere fusions) after escape (Supplementary Figure 2). These data are consistent with the JJN-3 DN-hTERT clones transiting a telomere erosion-induced crisis followed by eventual escape and re-immortalisation.

To examine whether PARPi affected the ability of JJN-3 DN-hTERT cells to escape a telomere crisis, we split a clonal JJN-3 DN-hTERT population (clone B) into twelve subpopulations before the onset of crisis (PD23), and cultured the cells in 1.0 μM, 2.5 μM, 5.0 μM and 7.5 μM of rucaparib, or, 0.5 μM. 1.25 μM, 2.5 μM and 3.75 μM of olaparib (Figure 2C, 2D). We also included untreated and DMSO-treated controls for each experiment. As expected, the untreated JJN-3 DN-hTERT cells entered crisis at around PD29 (day 42) and escaped after 17 days (day 59) to continue proliferating until PD53, when the experiments were terminated (Figure 2C). DMSO and low concentrations (1.0 μM and 2.5 μM) of rucaparib did not inhibit JJN-3 DN-hTERT cells from escaping crisis. However, cells treated with 5.0 μM and 7.5 μM of rucaparib failed to escape, managing only to divide up to PD33 and PD28, respectively (Figure 2C). These results were mirrored upon treatment with olaparib, where the highest concentrations used, 2.5 μM and 3.75 μM were sufficient to prevent JJN-3 DN-hTERT cells from escaping crisis (Figure 2D). We concluded that high concentrations of PARPi prevent JJN-3 DN-hTERT as well as HCT116 DN-hTERT cells from escaping crisis.

To confirm that the effects of PARPi on JJN-3 DN-hTERT cells were due to telomere dysfunction, we exposed a JJN-3 control population (transfected with an empty vector) to the same concentrations of rucaparib or olaparib for a similar period of time (Supplementary Figure 3). This control clone did not express DN-hTERT and so maintained its telomere length above that which could lead to a telomere-driven crisis. We observed that both rucaparib and olaparib slowed the rate of growth of JJN-3 cells (from 0.73 PD/day in the DMSO control to 0.42 PD/day in 7.5 μM rucaparib and 0.34 PD/day in 3.75 μM olaparib), but even at these concentrations JJN-3 cells were able to be propagated for up to 46 PDs and 42 PDs, respectively, at which point all the cultures were terminated. Overall, these results demonstrate that PARPi can inhibit human cells derived from both solid (HCT116) and haematological (JJN-3) malignancies from escaping a telomere crisis.

### PARPi do not affect the rate of telomere erosion but increase intra-chromosomal fusion in HCT116 DN-hTERT cells

**Figure 3:**
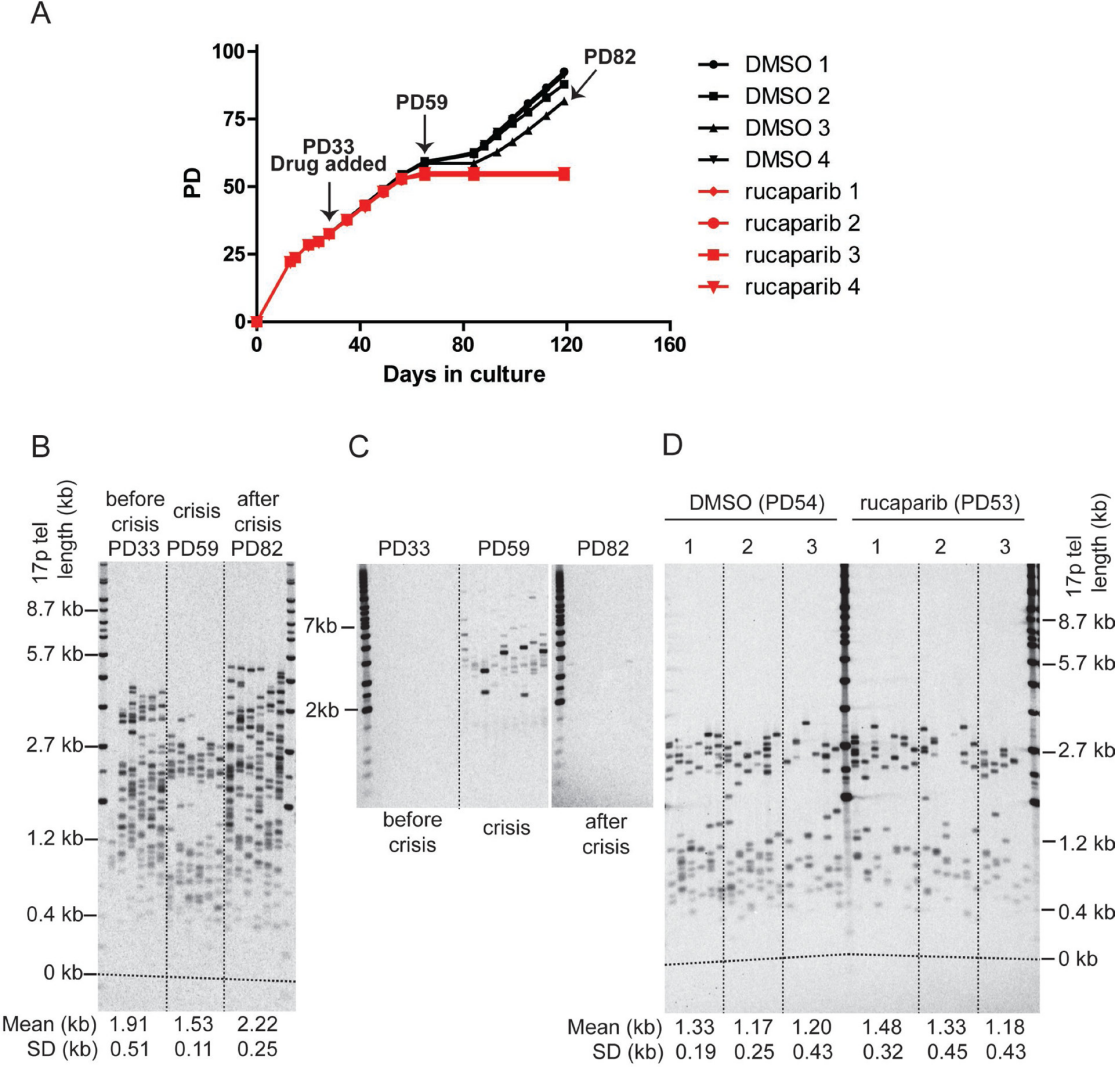
PARPi do not affect telomere erosion in HCT116 DN-hTERT cells during telomere crisis (**A**) Growth curve of HCT116 DN-hTERT cells treated with DMSO or 1µM of rucaparib. DMSO/rucaparib were added at the time indicated, and media/drug replaced at least once every three days until the termination of the experiment (PD = population doubling). (**B**) 17p STELA of HCT116 DN-hTERT cells (DMSO 3) at PD33 (before crisis), PD59 (crisis), and PD82 (escaped from crisis) (**C**) Telomere fusion analysis of HCT116 DN-hTERT cells (DMSO 3) at PD33 (before crisis), PD59 (crisis), and PD82 (escaped from crisis) using 17p, XpYp and 21q family telomere primers. Telomere fusion products were detected with a 17p telomere adjacent probe. (**D**) STELA of the 17p telomeres from cells treated with DMSO or 1 µM rucaparib at the indicated PD (*p* = 0.59, Mann–Whitney *U* Test, *n* = 3).

We next examined whether PARPi affect telomere dynamics during crisis. To do this, four independent HCT116 DN-hTERT cultures treated with 1 µM rucaparib were compared with four cultures treated with DMSO. We added rucaparib or DMSO to the cells at PD33 (∼22 PDs prior to crisis) and analysed telomere length distributions using STELA and telomere fusion using single-molecule PCR [30]. As we observed previously, cells treated with 1 µM rucaparib grew at a similar rate (0.72 PD/day) as the DMSO control cells (0.78 PD/day) and they entered crisis at approximately the same PD (∼PD53) and time point as the DMSO controls, but whilst all the DMSO-treated cells escaped crisis, none of the cell cultures treated with 1 µM rucaparib managed to survive (Figure 3A).

The telomere length distributions and fusions were examined in these cultures at PD33 (before crisis), PD59 (crisis), and (where possible) PD82 (escaped from crisis). As expected, telomere erosion was observed from an average length of 1.91 kb down to 1.53 kb prior to crisis (Figure 3B) and telomere fusions between the XpYp, 17p and 21q family telomeres was only detected during crisis (Figure 3C). Following the escape from crisis, the telomeres were elongated to an average length of 2.22 kb (Figure 3B); the telomere length distributions became more heterogeneous and the telomeres were stabilised as very few fusions could be detected in the post-crisis cells (PD82; Figure 3C). Thus, the telomere length and fusion profiles observed here are consistent with our previous observations of HCT116 DN-hTERT cells transiting a telomere erosion-induced crisis and escape following the re-establishment of telomerase activity [18].

We next assessed whether PARPi affected the rates of telomere erosion. We compared telomere length of DMSO- or rucaparib-treated cells at the point that the rucaparib-treated cells entered crisis (∼PD53), 28 days (∼20 PDs) after the addition of PARPi (Figure 3D). The telomeres of both group of cells were equally short and rucaparib did not have any significant impact on telomere length (*p* = 0.59, Mann–Whitney *U* Test). We concluded that PARPi do not affect telomere dynamics or impact on the ability of cells to escape telomere crisis by increasing the rate of telomere erosion.

**Figure 4:**
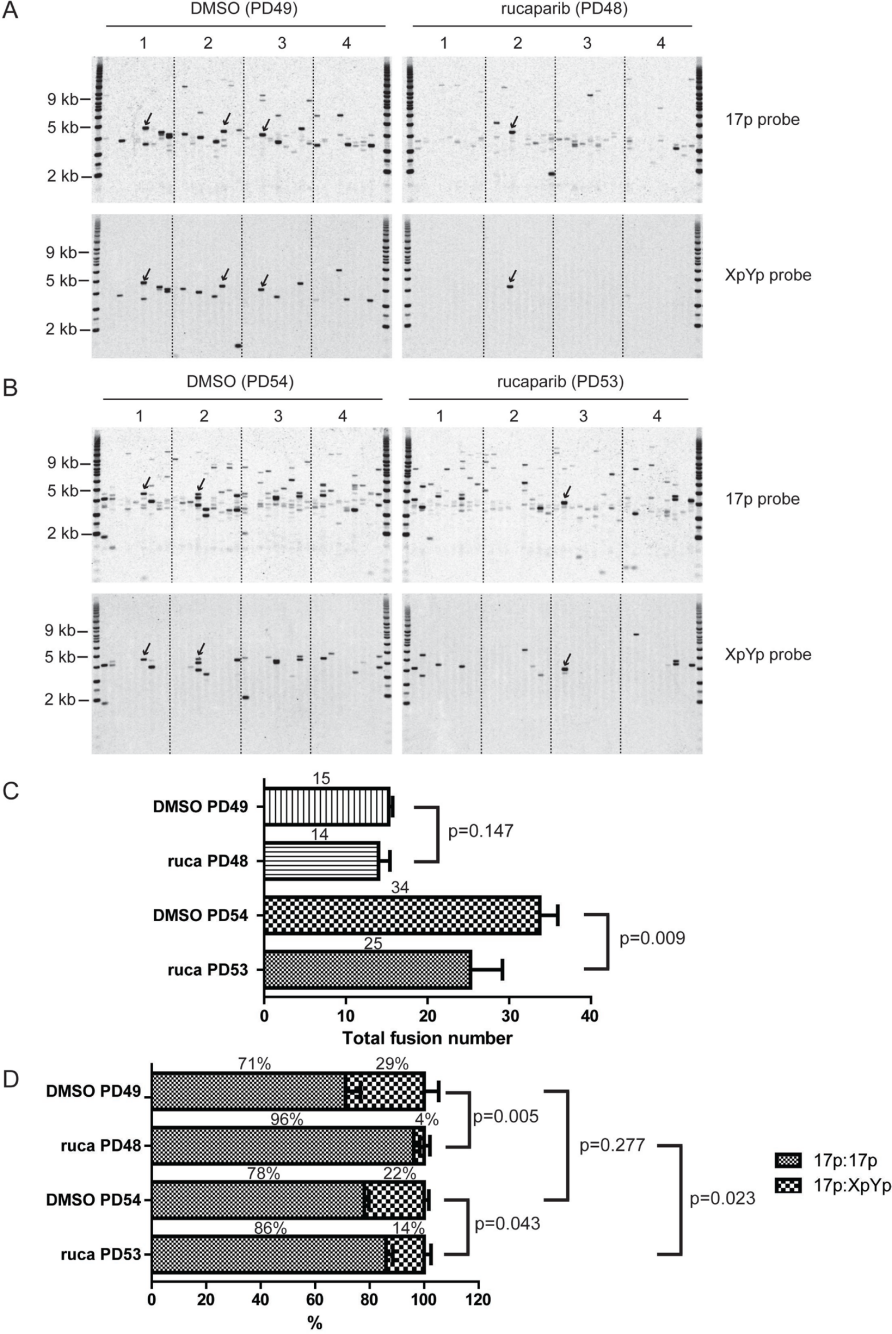
PARPi increases intra-chromosomal telomere fusion (**A**, **B**) XpYp:17p fusion analysis of HCT116 WT DN-hTERT cells treated with DMSO or 1 µM rucaparib at the indicated population doubling (PD). Telomere fusion were amplified using 17p and XpYp primers and detected with 17p or XpYp probes indicated on the right. Fusion bands detected with both probes are inter-chromosomal 17p: XpYp events (a few examples are indicated by arrows), whereas fusion detected with 17p probe only are intra-chromosomal 17p:17p events. (**C**, **D**) Bar chart showing quantification of total telomere fusion (**C**) or inter-chromosomal and intra-chromosomal fusion (**D**) in cells treated with DMSO or 1 µM rucaparib (ruca) at the indicated PD. The average number and proportion of telomere fusion are indicated on top of each bar. *P* values were obtained using Student’s *t*-test (2 tailed, equal variances, *n* = 4).

Our previous study indicated that the relative proportions of the inter-chromosomal, compared to intra-chromosomal telomere fusions, may impact on the ability of cells to escape crisis, with cells that exhibit a greater proportion of inter-chromosomal events being compromised in their ability to escape crisis, for example as observed in the context of LIG3-deficient cells [18]. To examine whether PARPi impacted the relative proportions of inter- and intra-chromosomal fusions, we compared the fusion of telomeres in cells treated with rucaparib or DMSO in our HCT116 DN-hTERT cells undergoing a telomere-driven crisis. We targeted the fusion assay to the XpYp and 17p telomeres, which allows inter- and intra-chromosomal fusion to be distinguished. At PD 48 to 49 (three weeks after the addition of PARPi/DMSO), we found evidence of both intra- (17p:17p) and inter- (17p:XpYp) chromosomal telomere fusion events and the total number of fusion is not significantly different between PARPi or DMSO treated cells (Figure 4A, 4C). However in contrast to that observed in the absence of LIG3 [18], there was a significant increase in intra-chromosomal 17p:17p fusion (96% vs 71%), accompanied by a reduction in inter-chromosomal 17p:XpYp fusion (4% vs 29%) in cells treated with PARPi (*p* = 0.005) (Figure 4A, 4D).

To further confirm this result, we examined telomere fusions in these cells at a later passage (PD 53 to 54) when the cells were deeper in crisis. As expected, we observed increased numbers of both 17p:17p and 17p:XpYp telomere fusion events in both the DMSO- and rucaparib-treated cells, as more telomeres were short and dysfunctional at this sampling point (Figure 4B, 4C). The total number of telomere fusion was reduced in PARPi treated cells compared to DMSO treated cell from 34 to 25 (*p* = 0.009) showing that PARPi affect fusion frequency deeper in crisis (Figure 4C). Consistent with the results in Figure 4A, rucaparib-treated cells displayed elevated intra-chromosomal 17p:17p fusion (86% vs 78%) and fewer inter-chromosomal 17p:XpYp fusion events (14% vs 22%) (*p* = 0.043; Figure 4B, 4D). Interestingly, the level of intra-chromosomal fusion significantly decreased (from 96% to 86%, *p* = 0.023, Figure 4D) in PARPi treated cells as these cells progressed through crisis, whereas the fusion spectrum was not significantly different in control cells between early and late passage (*p* = 0.277, Figure 4D). This observation suggests that some intra-chromosomal fusions elevated in PARPi treated cells may be deleterious, causing these cells to die thus reducing the number of total fusions observed in PARPi treated cells at late passage (Figure 4C). We concluded that the changes in the telomere fusion profiles observed in the context of PARPi were distinct from those observed in the absence of LIG3, which exhibited increased inter-chromosomal fusions [18]. Instead, PARPi treated cells displayed increased intra-chromosomal fusion which may be harmful to these cells.

### PARPi enhance DNA damage checkpoint activation and apoptosis in cells experiencing a telomere crisis

**Figure 5:**
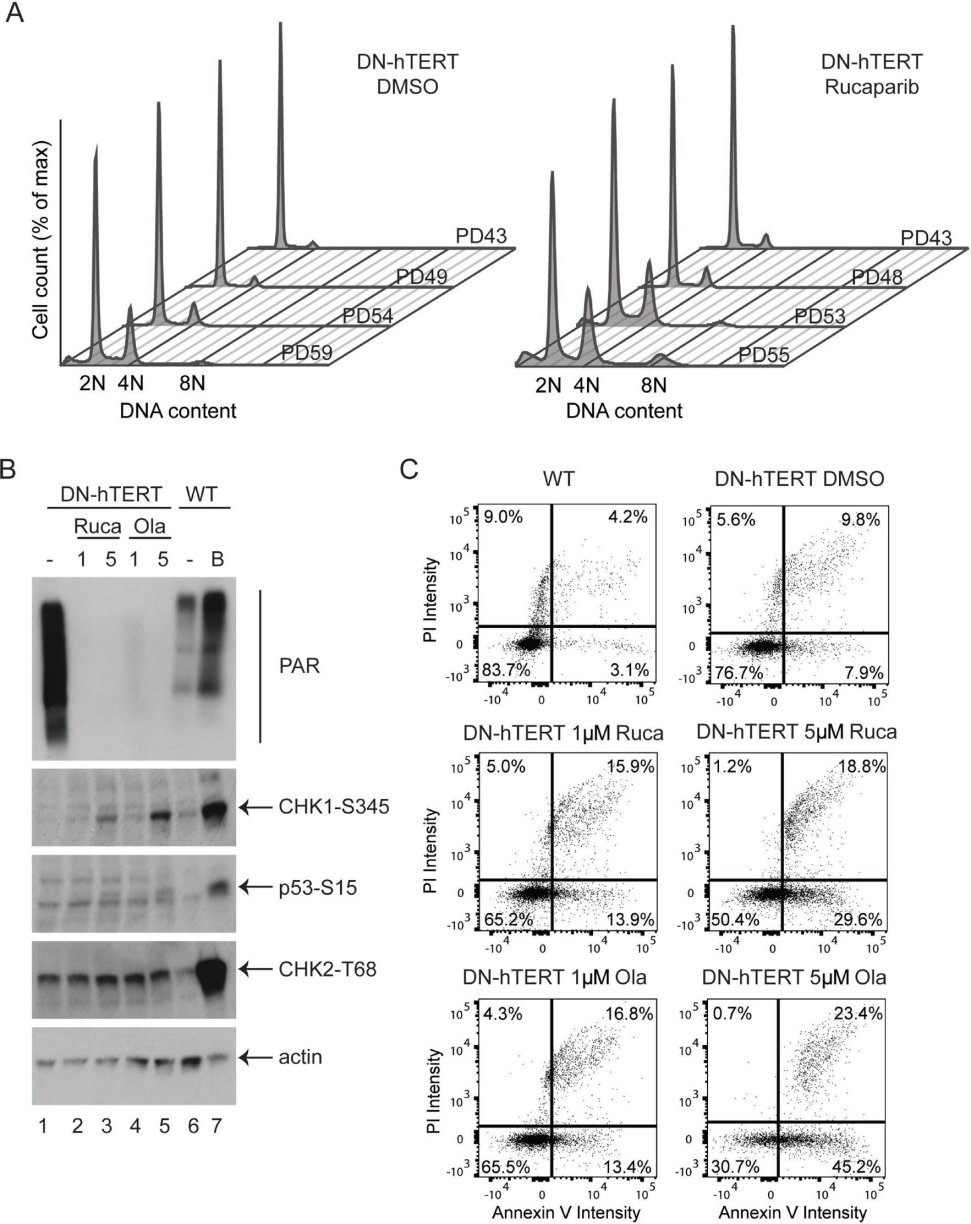
PARPi increases DNA damage checkpoint activation and apoptosis in HCT116 DN-hTERT cells (**A**) Cell cycle analysis of HCT116 WT DN-hTERT cells as they progress towards telomere crisis (PD59) in the presence of DMSO or 1 µM rucaparib. (**B**) Western blot analysis of a representative experiment from two showing the activation of various DNA damage markers (CHK1-S345, p53-S15 and CHK2-T68) in DN-hTERT cells after exposure to 1 µM or 5 µM of rucaparib (Ruca) or olaparib (Ola) for 15 days. HCT116 WT cells treated with a DNA damaging agent bleomycin (B) or untreated (–) were included as controls. The levels of PARylated proteins in these cells were also visualised using an anti-PAR antibody. (**C**) WT or DN-hTERT HCT116 (PD56) cells were exposed to PARPi for 15 days, and the level of apoptotic cells were quantified using Annexin-V/propidium iodide (PI) staining. One representative experiment from two is shown.

To further understand the effect of PARPi on cells undergoing a telomere-driven crisis, we also monitored the cell-cycle distribution of HCT116 DN-hTERT cells progressing through crisis in the presence of 1 µM rucaparib or DMSO (Figure 5A). As these cells progressed towards crisis, there was an increased accumulation of cells with a 4N or 8N DNA content (Figure 5A), supporting the observation that telomere damage induces tetraploidisation [31, 32]. Interestingly, rucaparib treatment significantly increased the proportion of cells with a 4N or 8N DNA content (Figure 5A and Supplementary Figure 4, *p* < 0.05 and *p* < 0.001 respectively, Student’s *T* test), suggesting that PARPi stimulate G2/M cell-cycle arrest in cells experiencing telomere crisis. As controls, we also examine HCT116 WT cells growing in the presence of 1 µM rucaparib or DMSO for a similar length of time (Supplementary Figure 5). We found that rucaparib did not strongly affect cell cycle progression in HCT116 WT cells (Supplementary Figure 5). We considered that the G2/M cell-cycle arrest observed in HCT116 DN-hTERT cells treated with rucaparib may be due to an enhanced DNA damage checkpoint response in cells experiencing a telomere crisis. To test this hypothesis, we examined the activation of CHK1, CHK2 and p53, which are three of the central DNA damage checkpoint effectors. HCT116 DN-hTERT cells at the onset of crisis (PD56) were exposed to 1 µM or 5 µM of rucaparib or olaparib and the phosphorylation of CHK1, CHK2 and p53 were monitored by western blot (Figure 5B). We also examined the level of PARylated proteins to confirm the activity of PARPi in these cells. As controls for DNA damage checkpoint activation, we included HCT116 wild type (WT) cells either not experiencing a telomere crisis (*i.e*., untreated) or cells treated with bleomycin (B) for 24 hr.

HCT116 DN-hTERT cells displayed high levels of PARylated proteins as shown by a smear detected with a PAR antibody (Figure 5B, lane 1). Consistent with the role of olaparib and rucaparib in inhibiting PARylation, both these drugs strongly reduced the level of detectable PARylated proteins (Figure 5B, compare lane 1 with lanes 2 to 5). As expected, bleomycin strongly activated the DNA damage response in WT HCT116 cells as CHK1, CHK2 and p53 were all heavily phosphorylated at their DNA damage induced phosphorylation sites (Figure 5B, compare lanes 6 and 7). Interestingly, both olaparib and rucaparib stimulated the phosphorylation of CHK1 and p53 in HCT116 DN-hTERT cells in crisis (Figure 5B, compare lane 1 with lanes 3 and 5), but no differences in CHK2 phosphorylation status were observed. We also examined the effect of PARPi in HCT116 WT cells and found that olaparib and rucaparib did not strongly activate CHK1 and p53 in these cells (Supplementary Figure 6). We concluded that PARPi synergise the DNA damage checkpoint activation by activating CHK1 and p53 in cells experiencing a telomere crisis.

As aberrant DNA damage checkpoint activation can stimulate apoptosis and cell death during a telomere-induced crisis, we next examined whether PARPi-induced DNA damage checkpoint activation enhanced apoptosis in HCT116 DN-hTERT cells. We used an Annexin V-PI assay to monitor apoptosis in WT HCT116 and HCT116 DN-hTERT cells which had been treated with 1 µM or 5 µM of rucaparib or olaparib and that were approaching crisis (Figure 5C and Supplementary Figure 7). WT HCT116 cell cultures had a low proportion (7.3%) of early (3.1%) and late stage (4.2%) apoptotic cells and PARPi increased these levels in WT HCT116 cells (1 µM rucaparib = 13.5%, 5 µM rucaparib = 16.2%, 1 µM olaparib = 12.5% and 5 µM olaparib = 23.3%; Supplementary Figure 7). As expected, HCT116 DN-hTERT cells (DMSO control) approaching crisis exhibited more apoptotic cells (9.8% + 7.9% = 17.7%) compared to WT HCT116 untreated cells (7.3%; Figure 5C). Importantly, both rucaparib and olaparib strongly stimulated apoptosis in these HCT116 DN-hTERT cells as the total level of apoptotic cells (1 µM rucaparib = 29.8%, 5 µM rucaparib = 48.4%, 1 µM olaparib = 30.2% and 5 µM olaparib = 68.6%) compared to the DMSO control (17.7%; Figure 5C) increased. We concluded that PARPi enhance DNA damage checkpoint activation and stimulate apoptosis in cells experiencing a telomere-induced crisis, and that this may account for why PARPi prevent the escape from a telomere crisis.

## DISCUSSION

Here we provide evidence that PARPi prevent the re-immortalization of human tumour cells by inhibiting their ability to escape from a telomere-driven crisis. We showed that PARPi treated cells accumulate more intra-chromosomal fusion during crisis. Cells treated with PARPi also exhibit enhanced DNA damage checkpoint activation and apoptosis. We believe that these phenotypes contribute to the ability of PARPi to prevent cells from escaping crisis.

PARPi are considered to act by primarily affecting PARP1 because: 1) PARP1 contributes to the majority of cellular PARP activity, and 2) PARPi have no effect on cellular DNA repair in the absence of PARP1 [33]. In addition to interfering with the activity of PARP1, PARPi physically trap PARP1 on DNA, which interferes with DNA repair and replication [34, 35]. PARPi such as rucaparib and olaparib can also inhibit the activities of other PARP family members to a lesser extent [36, 37]. However, our recent observation that deletion of *PARP1* also strongly prevents HCT116 from escaping a telomere crisis [38] suggest that instead of inducing PARP1 trapping or inhibiting other PARP family members, PARPi prevent escape from crisis mainly by inhibiting PARP1.

PARP1 participates in various pathways of DNA repair including DNA single-strand break repair (SSBR), double-strand break repair (DSBR), and replication fork repair [39]. Interestingly, we previously found that deletion of *LIG3*, a partner of PARP1 in the A-NHEJ pathway of DSBR, inhibits escape from a telomere crisis [18]. We proposed that a defect in A-NHEJ-induced intra-chromosomal fusion could be responsible for this observation [18]. However, PARPi do not lead to a similar defect in LIG3-induced intra-chromosomal fusion suggesting that the inability to escape crisis is likely not due to a A-NHEJ defect. In addition to A-NHEJ, LIG3 and PARP1 are also implicated together in the same pathway of SSBR [40]. Our previous analysis of *LIG3* mutants show that LIG3 requires its interaction with XRCC1, another protein involved in SSBR, to prevent escape from crisis, further implicating SSBR as an important factor in the escape from crisis [18]. A defect in SSBR could lead to accumulation of DNA single-stranded breaks and replicative stress in *LIG3* deficient or PARPi treated cells. We think that this would be particularly harmful in cells experiencing telomere crisis, since the telomeric regions are poorly protected by the Shelterin complex, especially its component TRF1, which is essential for the repair of stalled replication forks at telomeres to prevent ATR activation [41]. In support of this hypothesis, addition of PARPi to cells during crisis strongly activates Chk1 (Figure 5B), a downstream target of ATR which is induced by DNA single-stranded breaks and replicative stress [42]. We propose that this severe replicative stress could hyper-activate the DNA damage response in cells experiencing a telomere crisis to selectively killed PARPi treated or LIG3 deficient cells by stimulating apoptosis.

Alternatively, it is also possible that PARPi inhibit escape from a telomere crisis independently of LIG3. PARP1 has been implicated in telomere maintenance and PARP1 deficient cells have short telomeres and increased telomeric DNA damage [38, 43, 44]. It was shown recently that a PARP inhibitor (3-AB) inhibits the growth of pancreatic cancer cells treated with a telomerase inhibitor by inducing telomere shortening through the inhibition of Tankyrases [45]. However, we found that treatment of rucaparib did not induce telomere shortening in HCT116 DN-hTERT cells (Figure 3D). This is likely because rucaparib is a more selective inhibitor of PARP1 (compared to 3-AB) which only mildly affect the activities of Tankyrases [36]. PARP1 also interacts with the Shelterin component TRF2 and is recruited to eroded or damaged telomeres to facilitate DNA repair but the exact mechanism remains unclear [46, 47]. Recent studies show that PARP1 facilitates the recruitment of SLX4 complex to uncapped telomeres to initiate T loop cleavage and homologous recombination [48, 49]. We found that rucaparib treated cells cannot repair eroded telomeres in HCT116 DN-hTERT cells faithfully, as these cells accumulate more intra-chromosomal fusion (Figure 4). We speculate that PARPi treatment could reduce localisation of SLX4 complex and the homologous recombination machinery to eroded telomeres [48], causing a homologous recombination defect and an increase in sister telomere fusion (observed as intra-chromosomal fusion in Figure 4). Alternatively, PARPi could inhibit telomere repair by affecting TRF2 in a way that increases intra-chromosomal fusion [46]. These proposed defects in telomere repair could also contribute to the enhanced DNA damage checkpoint activation and apoptosis as observed in PARPi treated cells during crisis.

Even though PARP1 deletion in HCT116 DN-hTERT cells mirrors treatment with olaparib and rucaparib by strongly compromising cellular escape from a telomere crisis [38], we cannot rule out the possibility that inhibition of other members of PARP protein family by olaparib and rucaparib could also contribute to the ability of these drugs to inhibit crisis escape. Both olaparib and rucaparib could also inhibit PARP2, PARP3, PARP4, PARP10 and Tankyrases to a lesser extent than PARP1 [36, 37, 50], and these PARP family proteins have been implicated in DNA repair, telomere maintenance and mitosis [51–53]. It would be interesting to examine whether inhibitors that selectively inhibit these proteins also affect cellular escape from a telomere crisis [53–56].

Senescence and crisis represent two distinct proliferative lifespan barriers that are governed by replicative telomere erosion and they provide a tumour suppressive mechanism that must be overcome for progression to malignancy [1, 57]. While several drugs, called senolytic drugs, that selectively induce apoptosis of senescent cells have been identified [58], no drug that selectively eliminates cells undergoing a telomere-driven crisis have been described. Here we have shown that the two PARPi, olaparib and rucaparib, possess this property, which we term crisolytic, to allow them to prevent cellular escape from telomere crisis and inhibit cell re-immortalization.

One potential caveat from our study is that our experimental system utilises cancerous cell lines (HCT116 and JJN3) that are re-driven into the process of telomere crisis, escape and re-immortalisation. Thus, it would be important to examine whether crisolytic drugs have the same inhibitory effect on precancerous cells experiencing a telomere crisis in humans. Telomere erosion, dysfunction and fusion precede clinical progression in both solid and haematological cancers, and can be detected in very early stage lesions, providing evidence that a telomere crisis occurs early in the progression to malignancy [9, 10, 12, 59]. Importantly, telomere length accurately predicts the progression of cancer patients with early stage disease; for example, patients with CLL Binet stage A, who have short dysfunctional telomeres in their CLL B-cells, have a poorer prognosis and reduced overall survival [11, 14]. It would be interesting to test whether PARPi could selectively eliminate crisis cells with short dysfunctional telomeres from precancerous lesion or from these early stage patients.

Currently, PARPi are being used either as a chemotherapy potentiating agent, or as a monotherapy agent, in cancer patients who have cellular defect in homologous recombination-mediated DNA repair [23]. Several successful clinical trials have resulted in PARPi being approved for the treatment of advanced stage BRCA1/BRCA2-deficient ovarian cancer patients that have been pre-treated with chemotherapy, or who have undergone surgery [23]. However, most cancer cells do not exhibit these features and there are concerted efforts to identify biomarkers to stratify patients that would be responsive to PARPi treatment [26]. Our data support further preclinical and clinical evaluation of PARPi as a potential treatment for cancer patients with tumours that exhibit short dysfunctional telomeres, or for the treatment of tumours in combination with drugs that induce telomere dysfunction (such as inhibitors of telomerase or ligands of G quadruplex DNA) as demonstrated by others [45, 47]. Importantly, we found that PARPi can selectively eliminate cells in crisis that are either p53-positive (HCT116) or p53-negative (JJN-3). In the case of p53-negative cells, we propose that PARPi stimulates a p53-independent DNA damage response and apoptosis in these cells, as JJN-3 cells are fully capable of activating a robust DNA damage response and apoptosis following DNA damage [60]. Thus, we propose that PARPi has the potential to target a wide range of tumours with dysfunctional telomeres regardless of p53 status.

In summary, we provide the first demonstration that clinically approved drugs that inhibit PARP1 can selectively target and eliminate cells experiencing a telomere crisis to prevent cell proliferation and immortalisation, suggesting the exciting possibility of novel therapeutic avenues for PARPi in the context of tumours and pre-cancerous lesions exhibiting short dysfunctional telomeres.

## MATERIALS AND METHODS

### Cell culture and analysis

HCT116 (WT and DN-hTERT) human colorectal carcinoma cell lines were as described [18] and grown in McCoy’s 5A medium supplemented with 10% fetal calf serum (FCS). JJN-3 cells were cultured in Dulbecco’s Modified Eagle Medium (DMEM) supplemented with sodium pyruvate (1 mM - Invitrogen), penicillin (100 Units/ml - Sigma), streptomycin (0.1 mg/ml - Sigma), non-essential amino acids (1X - Sigma), FCS (20% v/v - Thermo Fisher Scientific) and L-glutamine (2 mM - Sigma). Telomere crisis experiments were started from clonal cells frozen at different PDs. Cell cycle analyses were performed using a two-step cell cycle protocol on a NucleoCounter NC-3000™ system (Chemometec). Apoptosis was assessed using an Annexin V-FITC apoptosis detection kit (eBioscience) on a NucleoCounter NC-3000™ system (Chemometec). Rucaparib (S1098-SEL) and olaparib (S1060-SEL) were purchased from Stratech Scientific (UK).

### STELA, telomere fusion assay and TRAP

17p and XpYp STELA were performed to determine telomere length according to standard protocols [18, 30]. Briefly, genomic DNA was isolated using standard phenol/chloroform protocol and diluted to 10 ng/μL in 10 mM Tris-HCl (pH 7.5). Ten nanograms of DNA were further diluted to 250 pg/μL in a volume of 40 μL containing 1 μM Telorette2 linker and 1 mM Tris-HCl (pH 7.5). 1 μL of this DNA/Telorette 2 solution were subjected to PCR in a 10 μL reaction containing 0.5 μM telomere-specific primers, 0.5 μM Teltail primer and 0.5 U of a 10:1 mixture of Taq (Thermo Fisher Scientific) and Pwo polymerase (Roche). The PCR products were resolved by 0.5% TAE agarose gel electrophoresis and were detected by Southern hybridization with a random-primed α-^33^P-labeled (GE Healthcare) TTAGGG repeat probe. Telomere fusion assay were performed as described [30, 61]. Briefly, 50 ng of phenol/chloroform extracted DNA were subjected to PCR in a 10 μL reaction containing 0.5 μM telomere-adjacent primers (XpYpM, 17p6 and 21q1) and 0.5 U of a 10:1 mixture of Taq (Thermo Fisher Scientific) and Pwo polymerase (Roche). Fusion molecules were detected by Southern blotting as described above and detected with a XpYp or 17p telomere-adjacent probes. Telomerase activity was quantified using the TRAPeze XL Telomerase detection kit (Chemicon International).

### Cell lysis and western blot analyses

Cells were lysed in lysis buffer (150 mM NaCl, 50 mM Tris HCl, 5 mM EDTA, 1% NP40, 3 mM PMSF, 1/100 protease inhibitor cocktail III [Calbiochem 539134] and 1/100 phosphatase inhibitor cocktail II [Calbiochem 524625]) on ice for 5 min and centrifuged at 20,000g for 30 min. The proteins in the supernatant were removed and quantified using a Pierce Coomassie plus protein assay reagent (23236, Thermo Fisher Scientific). For western blot analyses, proteins were separated on 7.5% Mini-PROTEAN TGX™ precast protein gels (456-1026, Biorad), transferred to PVDF membranes (Millipore) and probed with either an anti-PAR rabbit polyclonal antibody (4336-BPC-100, Trevigen), an anti-phospho-Chk1 (Ser345) rabbit monoclonal antibody (2348, Cell signalling), an anti-phospho-p53 (Ser15) mouse monoclonal antibody (9286, Cell signalling), an anti phospho-Chk2 (Thr68) rabbit polyclonal antibody (2661, Cell signalling) or an anti-actin rabbit polyclonal antibody (A2066, Sigma-Aldrich).

## SUPPLEMENTARY MATERIALS FIGURES



## Abbreviations

PARPi: PARP inhibitors
LIG3: DNA ligase III
LIG4: DNA ligase IV
A-NHEJ: alternative non-homologous end-joining
C-NHEJ: classical non-homologous end-joining
PARylation: poly-ADP-ribosylation
DN-hTERT: dominant negative human telomerase
PDs: population doublings
DMSO: dimethyl sulfoxide
STELA: Single telomere length analysis
SSBR: DNA single-strand break repair
DSBR: DNA double-strand break repair
