# Supplementary material for: PARP inhibition prevents escape from a telomere-driven crisis and inhibits cell immortalisation
Source: Oncotarget. 2018 Dec 25;9(101):37549–63. doi: 10.18632/oncotarget.26499 (PMC6331021; doi:10.18632/oncotarget.26499)
Supplement: Supplementary file 1 [file oncotarget-09-37549-s001.pdf]

PARP inhibition prevents escape from a telomere-driven crisis and inhibits cell immortalisation

SUPPLEMENTARY MATERIALS

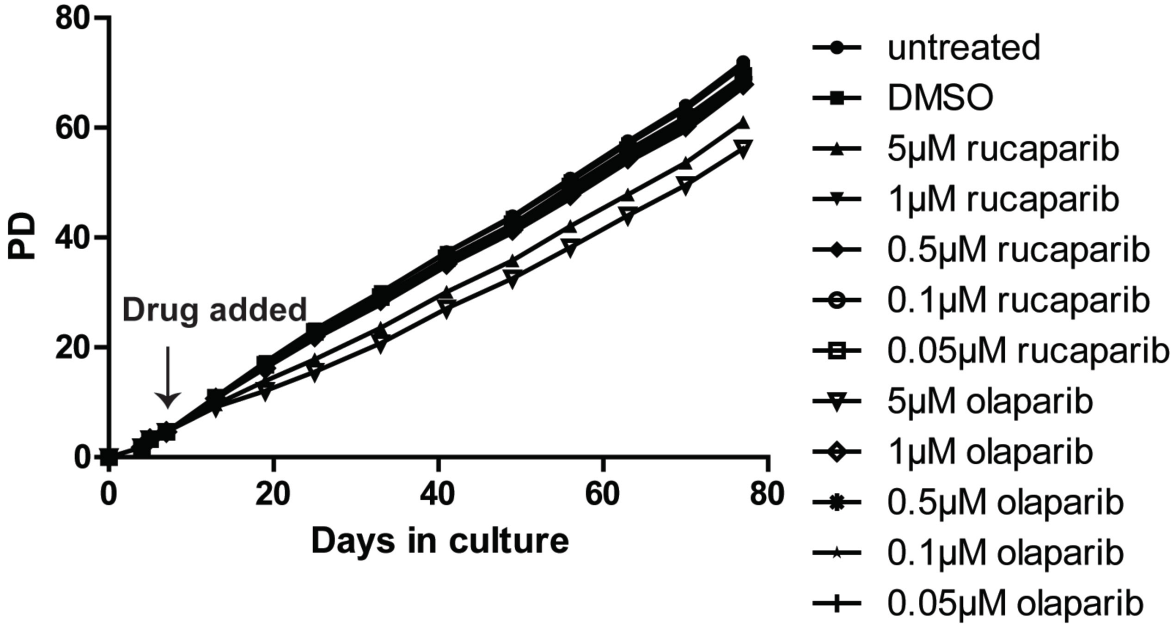

Supplementary Figure 1: Growth curve of HCT116 WT cells treated with the indicated concentration of rucaparib or olaparib. Each PARPi was added at the time indicated, and media/drug replaced at least once every three days until the termination of the experiments. (PD = population doubling).

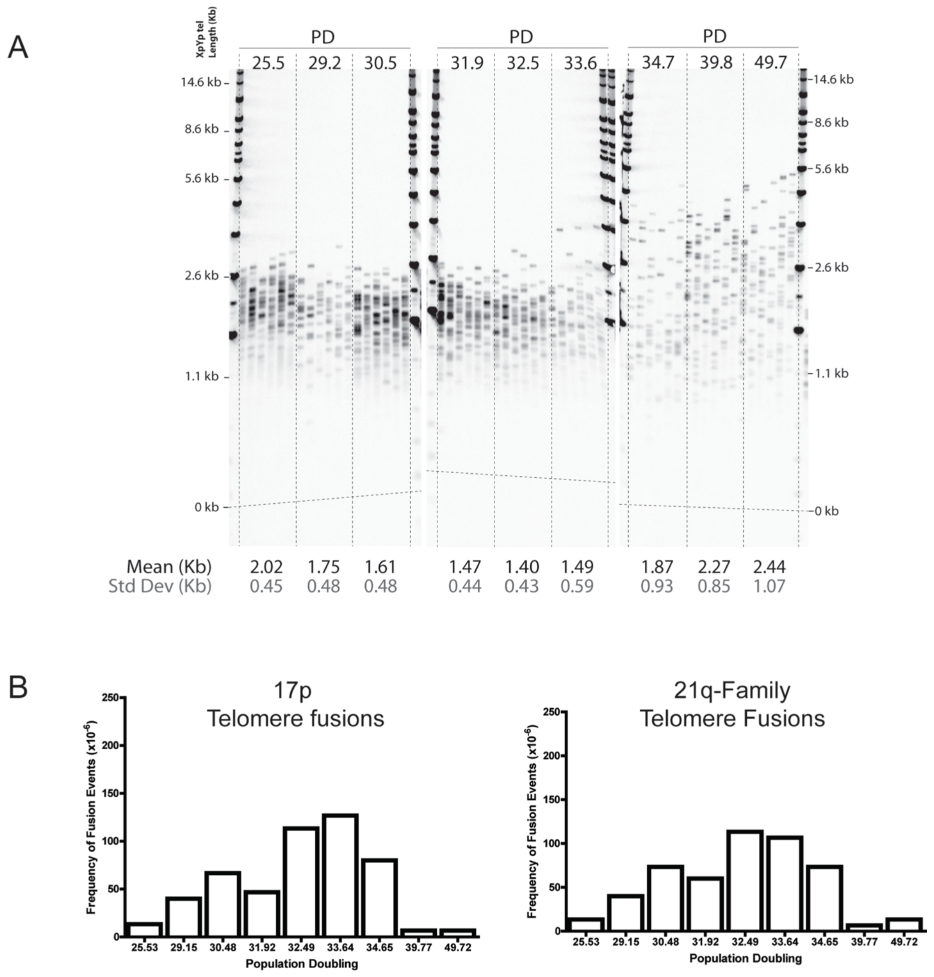

Supplementary Figure 2: (**A**) STELA profile showing the XpYp telomere lengths, during a period of telomere-driven crisis and escape, in a clonal population of JJN-3 cells expressing DN-hTERT. (PD = population doubling). (**B**) Graphs showing the frequency of telomere fusion events involving either the 17p or 21q-family of chromosome ends. The frequency of chromosomal fusion was estimated by dividing the number of observable fusion bands by the number of input molecules.

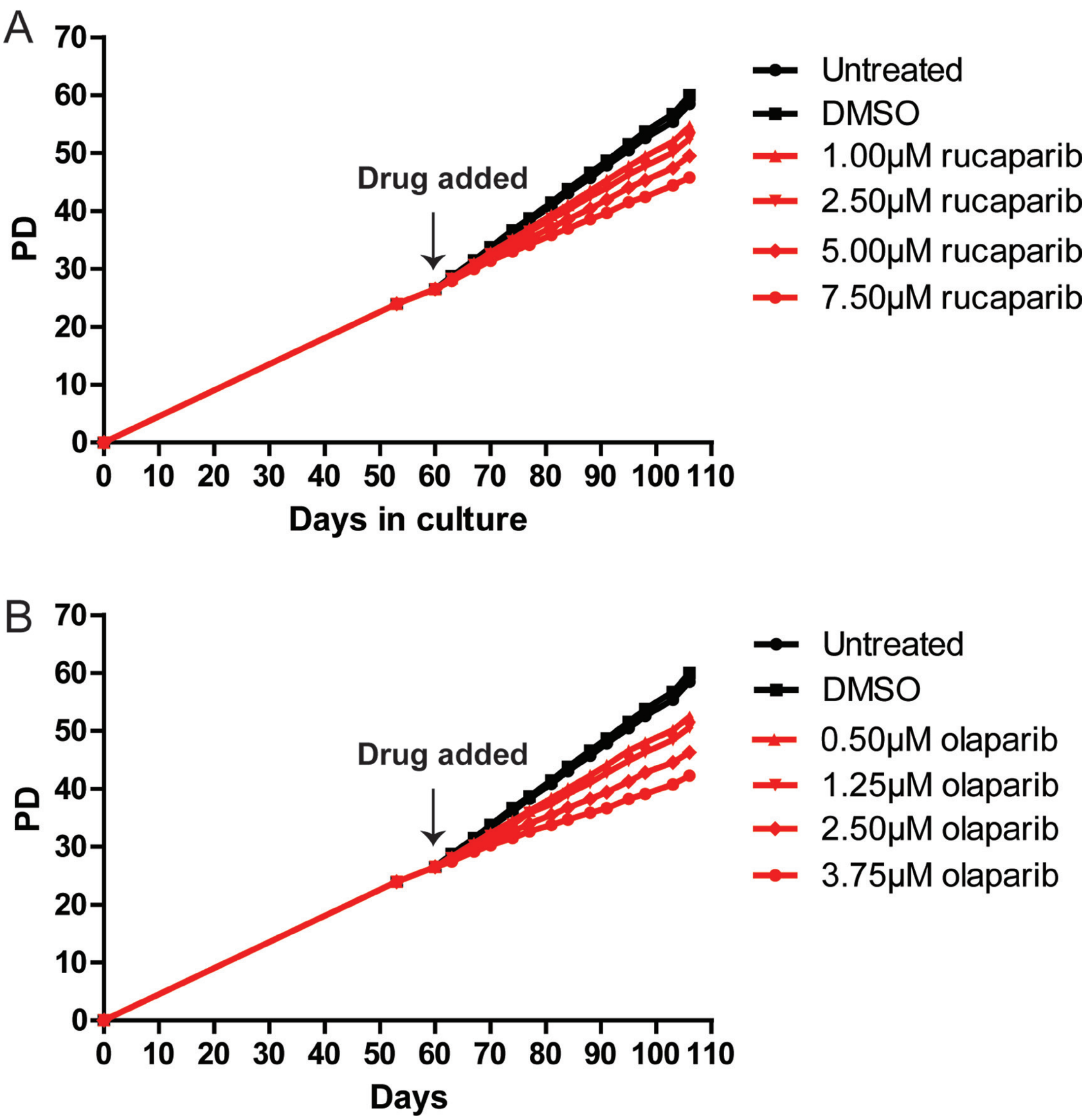

Supplementary Figure 3: (**A**, **B**) Growth curve of JJN-3 WT cells treated with the indicated concentrations of olaparib and rucaparib. Each PARPi was added at the time indicated, and media/drug replaced at least once every three days until the termination of the experiments. The untreated and DMSO controls are the same in (**A** and **B**).

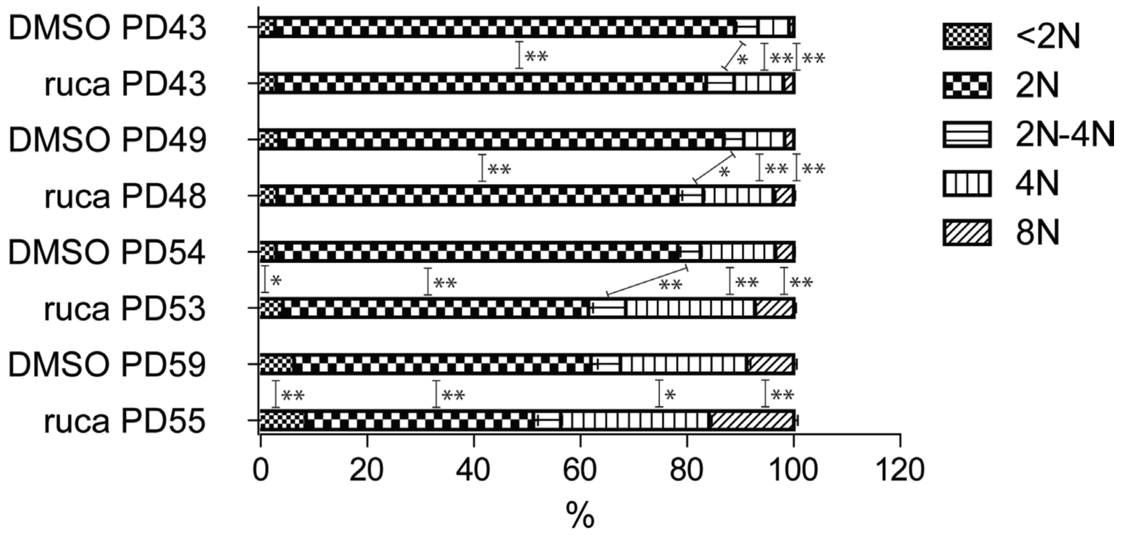

Supplementary Figure 4: Bar chart showing quantification of cells in different cell cycle stages at the indicated PD from four independent cultures of 1µM rucaparib (ruca) and DMSO treated cells (Figure 3A). *P* values were obtained using Student's t-test (2 tailed, equal variances, $n = 4$). *indicates $p < 0.05$, **indicates $p < 0.001$.

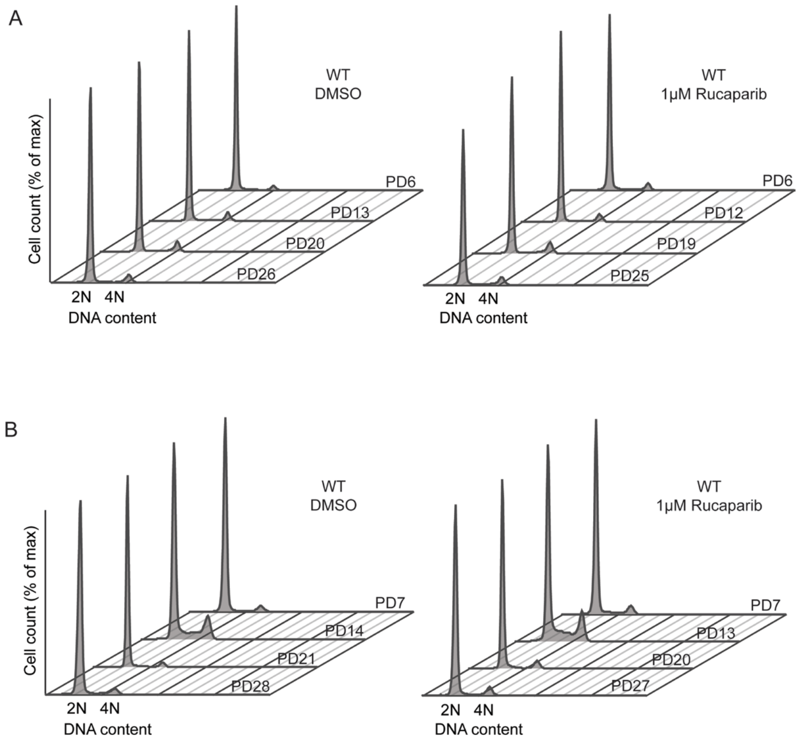

Supplementary Figure 5: (**A**, **B**) Cell cycle analysis of HCT116 WT cells (**A** = a clonal population of HCT116 carrying a control empty vector, **B** = parental HCT116) growing in the presence of DMSO or 1 μM rucaparib. (PD = population doubling).

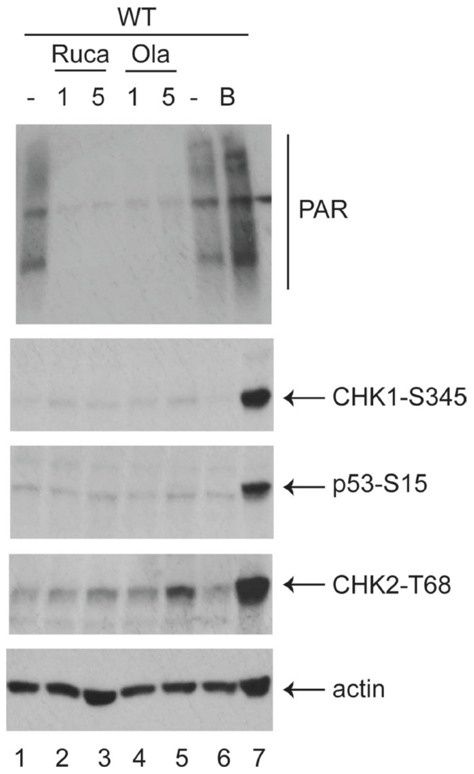

Supplementary Figure 6: . Western blot analysis of a representative experiment from two showing various DNA damage markers (CHK1-S345, p53-S15 and CHK2-T68) in HCT116 WT cells after exposure to 1 µM or 5 µM of rucaparib (Ruca) or olaparib (Ola) for 15 days. HCT116 WT cells treated with a DNA damaging agent bleomycin (B) or untreated (–) were included as controls. The levels of PARylated proteins in these cells were also visualised using an anti-PAR antibody.

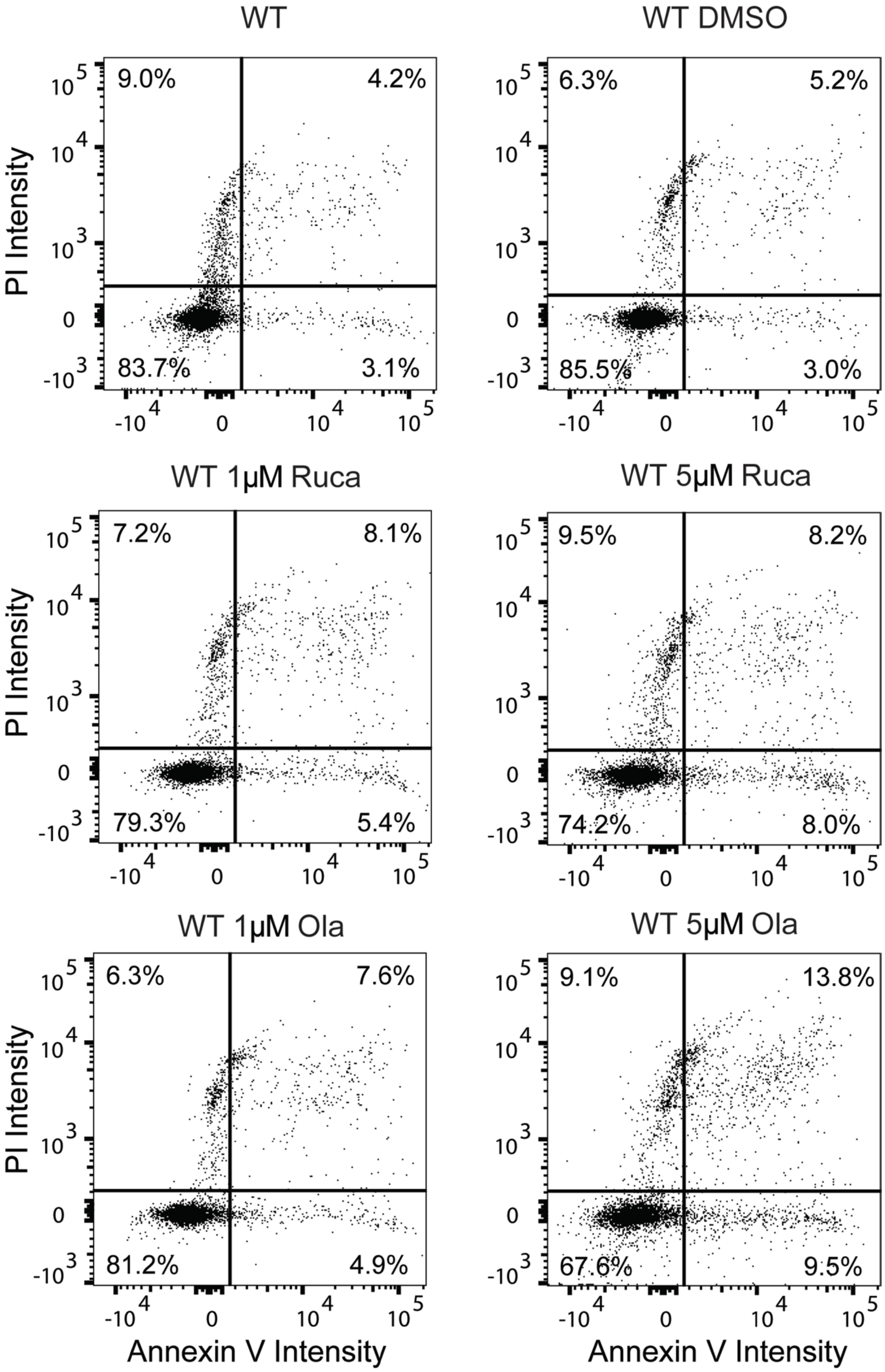

Supplementary Figure 7: HCT116 WT cells were exposed to PARPi for 15 days and the level of apoptotic cells were quantified using Annexin-V/propidium iodide (PI) staining. The untreated WT culture is the same control as in Figure 5C.
